# Five Antigen Tests for SARS-CoV-2: Virus Viability Matters

**DOI:** 10.3390/v13040684

**Published:** 2021-04-15

**Authors:** Miroslav Homza, Hana Zelena, Jaroslav Janosek, Hana Tomaskova, Eduard Jezo, Alena Kloudova, Jakub Mrazek, Zdenek Svagera, Roman Prymula

**Affiliations:** 1 Hospital Karvina-Raj, Vydmuchov 399, 734 01 Karvina, Czech Republic; mirek.homza@centrum.cz; 2 Department of Internal Medicine, Faculty of Medicine, University of Ostrava, Syllabova 19, 703 00 Ostrava, Czech Republic; 3 Institute of Public Health Ostrava, Partyzánské Náměstí 7, 702 00 Ostrava, Czech Republic; hana.tomaskova@zuova.cz (H.T.); eduard.jezo@zuova.cz (E.J.); alena.kloudova@zuova.cz (A.K.); jakub.mrazek@zuova.cz (J.M.); 4 Department of Biomedical Sciences, Faculty of Medicine, University of Ostrava, Syllabova 19, 703 00 Ostrava, Czech Republic; zdenek.svagera@fno.cz; 5 Faculty of Medicine, University of Ostrava, Syllabova 19, 703 00 Ostrava, Czech Republic; janosek@correcta.cz; 6 Department of Epidemiology and Public Health, Faculty of Medicine, University of Ostrava, Syllabova 19, 703 00 Ostrava, Czech Republic; 7 Department of Clinical Biochemistry, Institute of Laboratory Medicine, University Hospital Ostrava, 17. Listopadu 1790/5, 708 00 Ostrava, Czech Republic; 8 Faculty of Medicine Hradec Kralove, Charles University Prague, Simkova 870, 500 03 Hradec Kralove, Czech Republic; prymula@seznam.cz

**Keywords:** SARS-CoV-2, COVID-19, RT-PCR, antigen testing, infectiousness, viability

## Abstract

Antigen testing for SARS-CoV-2 (AGT) is generally considered inferior to RT-PCR testing in terms of sensitivity. However, little is known about the infectiousness of RT-PCR positive patients who pass undetected by AGT. In a screening setting for mildly symptomatic or asymptomatic patients with high COVID-19 prevalence (30–40%), 1141 patients were tested using one of five AGTs and RT-PCR. Where the results differed, virus viability in the samples was tested on cell culture (CV-1 cells). The test battery included AGTs by JOYSBIO, Assure Tech, SD Biosensor, VivaChek Biotech and NDFOS. Sensitivities of the ATGs compared to RT-PCR ranged from 42% to 76%. The best test yielded a 76% sensitivity, 97% specificity, 92% positive, and 89% negative predictive values, respectively. However, in the best performing ATG tests, almost 90% of samples with “false negative” AGT results contained no viable virus. Corrected on the virus viability, sensitivities grew to 81–97% and, with one exception, the tests yielded high specificities >96%. Performance characteristics of the best test after adjustment were 96% sensitivity, 97% specificity, 92% positive, and 99% negative predictive values (high prevalence population). We, therefore, believe that virus viability should be considered when assessing the AGT performance. Also, our results indicate that a well-performing antigen test could in a high-prevalence setting serve as an excellent tool for identifying patients shedding viable virus. We also propose that the high proportion of RT-PCR-positive samples containing no viable virus in the group of “false negatives” of the antigen test should be further investigated with the aim of possibly preventing needless isolation of such patients.

## 1. Introduction

RT-PCR testing is currently considered the most reliable technique for identification of patients infected with the new coronavirus SARS-CoV-2. However, antigen tests from nasopharyngeal swabs (AGTs) providing rapid response at the point-of-care are also deemed suitable for screening purposes [1]. In a screening setting, both of these methods usually use nasopharyngeal swabs (NPS), although oropharyngeal swabs are sometimes used or combined with NPS. The sensitivity of antigen tests related to RT-PCR varies, depending on the brand and setting of the evaluation experiment, e.g., [1,2,3,4,5,6]. For example, the cycle threshold considered as the cut-off for SARS-CoV-2 positivity plays a major role in the sensitivity testing, with lower cycle threshold values corresponding to higher viral loads and, inherently, better AGT performance.

However, few studies take into account the viability of the virus detected by RT-PCR, which is associated with infectiousness (in particular, in patients in the early stages of the disease or only mild symptoms) [7,8,9]. We hypothesized that in a significant proportion of patients in whom RT-PCR detected SARS-CoV-2 but who were tested negative by AGT, only non-viable virus is present on the mucosa. For this reason, we evaluated five AGTs using RT-PCR as the reference standard and supplemented it with an assessment of virus viability using cell culture in samples where RT-PCR and AGT tests differed.

## 2. Materials and Methods

### 2.1. Patient Group and Test Setting

The study was approved by the local Ethics Committee, No. NsPKar/19956/2020/SEK. All patients signed informed consent prior to inclusion in the study.

The tests were compared in a real-world high-throughput setting of asymptomatic (48.3%) and/or mildly symptomatic (51.7%) patients. Consecutive individuals referred to our testing point due to a contact with a SARS-CoV-2 positive person or due to having COVID-19-like symptoms with course not requiring hospitalization were included in the study. Two (one from each nostril) nasopharyngeal swabs were taken from each patient by trained medical personnel. One swab was placed into the transport medium (D-MEM, 0.5% bovine serum albumin), the other into the medium supplied with the respective AGT (one AGT per person was performed). The AGT was performed immediately, while RT-PCR and, if necessary, viability tests were performed after the sample was transported into the laboratory. The tests were performed within 24 h of sampling; where this was not possible, samples were frozen at −80 °C and thawed immediately before testing.

### 2.2. Antigen Testing

Five antigen tests, namely SARS-CoV-2 Antigen Rapid Test Kit (JOYSBIO (Tianjin) Biotechnology Co., Ltd., Tianjin, China); Ecotest COVID-19 Antigen Rapid Test (Assure Tech, Hangzhou, China; not to be mistaken for Eco Test by ECO Diagnostica, Nova Lima, Brazil); Standard Q COVID-19 Ag (SD Biosensor, Korea); Immupass VivaDiag™ SARS-CoV-2 Ag Rapid Test (VivaChek Biotech (Hangzhou) Co. Ltd., Hangzhou, China); and ND COVID-19 Ag test (NDFOS, Eumseong, Korea) were provided to us for evaluation by the Ministry of Health of the Czech Republic. Testing was performed according to the manufacturers’ instructions.

### 2.3. RT-PCR

RT-PCR analysis was performed using the Automated RNA Isolation Kit for Agilent Bravo (DIANA Biotechnologies, Vestec, Czech Republic) and the PCR detection kit COVID-19 Multiplex RT-PCR Kit (DIANA Biotechnologies, Czech Republic) according to the manufacturer’s instructions. The isolation procedure resulted in an approx. 3× increase in RNA concentration. The detection was based on the proof of two SARS-CoV-2 genes, namely genes coding the spike protein and endoRNAse. The entire process was controlled through isolation and amplification of a synthetic internal control. Cycle thresholds (Ct) for both genes were recorded and the mean Ct from each sample was included in the analysis. Samples were evaluated as Strongly positive (Ct ≤ 20), Positive (20 < Ct ≤ 30), Weakly positive (30 < Ct ≤ 40), and Negative (CT > 40). The reference standard supplied by the PCR kit manufacturer containing 2 × 10^6^ RNA copies/mL sample was detected at Ct 26. By calculation, the Ct cut-offs for individual groups are approximately 1.28 × 10^8^ (Ct = 20) and 1.25 × 10^5^ (Ct = 30) RNA copies/mL.

### 2.4. Virus Viability Testing

Virus viability was assessed only in samples where the result differed between the antigen test and RT-PCR. Testing was performed in monolayer CV-1 cells (African green monkey kidney fibroblasts) cultured at 37 °C in the E-MEM medium (Sigma-Aldrich, St. Louis, MO, USA) in Leighton tubes. Cells were inoculated with 300 µl of the sample used for the RT-PCR testing or blanks. The cultures were examined daily under the microscope (100–200× magnification) for changes indicating a cytopathic effect of the virus. After 7 days (or sooner if the cytopathic effect was observed in approx. 75% of cells), the cells were passaged (1:6) and cultured for an additional 7 days. If no cytopathic effect (virus action) was observed over the next 7 days, the sample was declared free of viable virus. Where cytopathic effect was observed, SARS-CoV-2 presence was verified by RT-PCR.

The sensitivity of the method was verified through serial dilution of the virus stock suspension prepared by cultivation and measured by PCR (3 × 10^11^ RNA copies/mL), both directly and after freezing at −80 °C. The cytopathic effect was observed from approx. 10^4^ RNA copies/mL, which, with the infectious particles:RNA ratio in the tissues ranging typically between 1:1000 and 1:10,000 [10,11] corresponds to 1–10 infectious particles/mL.

### 2.5. Statistical Analysis

Antigen test parameters (sensitivity, specificity, positive and negative predictive values, test accuracy) were calculated in Stata v.14 (https://www.stata.com/stata14/) and Clinical Calculator 1 (http://vassarstats.net/clin1.html) comparing the AGT results to the result of a) RT-PCR only and b) RT-PCR corrected on the viability in discrepant samples (where PCR test was positive but no viable virus was present, samples were considered negative). 95% confidence intervals were calculated for all parameters. The dataset is available from Figshare at https://doi.org/10.6084/m9.figshare.14096648.v1.

## 3. Results

In total, 1141 patients were tested within this study. The numbers of patients, their mean ages, percentages of symptomatic patients, and sex representations are shown in Table 1.

Out of these 1141 patients, 734 (65.2%) were SARS-CoV-2 negative according to RT-PCR, 193 samples (16.9%) were weakly positive (30 < Ct ≤ 40), 196 (17.2%) positive (20 < Ct ≤ 30), and 8 (0.7%) strongly positive (Ct ≤ 20). Most discrepant results between AGTs and RT-PCR were detected, as expected, in the group of patients with weak RT-PCR positivity (81.4% of discrepant results). All patients in the patient group with strong RT-PCR positivity were detected also by the AGTs. More details are shown in the Appendix A, Table A1.

Table 2 details the overall agreement between RT-PCR and individual antigen tests as well as false positive and false negative results relative to both selected reference methods. It is obvious that while Ecotest and Standard Q tests yielded relatively good results even when compared to RT-PCR itself, the performance of other tests, in particular ND COVID, was not as good, primarily due to a high number of false-positive results. It was the only test with such a high false-positive rate. In all, viable virus was present only in 14.1% of the samples with the false-negative result (AGT compared to RT-PCR).

Performance parameters of individual tests in combination with the prevalence of SARS-CoV-2 positive samples in the screening setting are detailed in Table 3. Compared to PCR only, none of the tests returned the target 90% sensitivity set by the European Centre for Disease Control [1]. However, when considering RT-PCR positive samples containing no viable virus as negative on the grounds that these patients do not pose any risk of spreading the virus, the required performance was met by two tests (with the best values, i.e., sensitivity of 96.4% and specificity of 97%, recorded for the Ecotest by AssureTech) and two more tests were very close. However, one of these, ND COVID, failed to meet the specificity requirements, with specificity both before and after including virus viability in the testing at approx. 60%.

Table 4 and Table 5 detail the results separately for symptomatic and asymptomatic patients, respectively. Please note that for several patients, symptomatic status was not self-reported; such patients were removed from analysis (Ecotest—5 patients; JoysBio—6 patients; remaining tests—1 patient each). In symptomatic patients, all tests met the WHO criteria for sensitivity; one test (Ecotest) achieved even as much as 98.6% sensitivity. Most tests performed relatively well even in the identification of asymptomatic PCR patients with viable virus; however, the numbers of such patients in our patient group were very low and, hence, the results can be considered rather as an indication of good performance than as its proof.

## 4. Discussion

In this study, we have compared five antigen tests (AGTs) for detection of SARS-CoV-2 in nasopharyngeal swabs and where the results of RT-PCR and AGT disagreed, we performed viability testing. As far as we know, the presented study is the largest one evaluating the virus viability in association with the antigen testing in a real-world high-prevalence setting so far. As expected, the highest number of discrepant results was in the group of patients with high Ct numbers (weak positivity), with approx. 70% of all discrepancies falling within these groups. The only exception was the ND COVID test with a very high false-positive rate of 44%.

Relative to RT-PCR only, all tests performed significantly poorer in our study than reported by the manufacturers. This is true even for Standard Q, verified by multiple studies [5]. However, it is necessary to say that in those studies, the share of symptomatic patients was very high (84.6–99.8%) and the Ct values for RT-PCR positivity were set lower [5] than in our study (40 cycles), which necessarily leads to the exclusion of the majority of samples with discrepant results and, in effect, to the improvement in sensitivity. Results from a study without such population preselection (i.e., a study that was closer to the real-world high throughput setting used in this paper) reported a sensitivity of 70% [12], which is much closer to our results than those reported in [5]. Besides, Standard Q was the test with the lowest number of samples in our study (caused by the number of tests provided to us by the Ministry) as well as the lowest number of positive samples so the result is the least reliable of all tests. Ecotest by AssureTech yielded the best results both before and after the correction on viability. After correcting on viability, the JOYSBIO test also performed very well (sensitivity over 92%) followed by Standard Q. The VivaDiag test closely met the WHO criteria of 80% sensitivity. As mentioned above, the ND COVID test performed poorly in particular because of the high false-positive rate. Except for the ND COVID test, all other tests yielded also very good results in another parameter of utmost importance for prevention of disease spreading, i.e., the negative predictive value (which is, unfortunately, rarely reported). Negative predictive values after correction on viability, i.e., the probability that the patient indeed poses no threat of disease transmission when negatively tested, was over 96% in the four better-performing tests (and in the case of Ecotest, as much as 98.7%), even in a high prevalence setting.

Regardless of the performance of the individual tests, the most important outcome of our study is the generally very low rate of viable virus presence in the samples which are conventionally considered to be “false negatives of the antigen tests,” which was particularly true for the samples with weakly positive RT-PCR results (Ct > 30; see Table A1 in Appendix A). This suggests that most asymptomatic or mildly symptomatic patients who are SARS-CoV-2 positive according to RT-PCR but missed by the evaluated AGTs (at least the better-performing ones) are probably not infectious. In this respect, tests performing best in our study when corrected on viability appear to be an extremely valuable tool for the identification of patients capable of spreading viable virus in the high throughput regime. Theoretically, if confirmed by further studies, this could in the long-term potentially lead even to replacement of high-volume RT-PCR testing in a high prevalence setting by well-performing antigen tests. Such studies would need to take into account, in particular, temporal development of all parameters observed in our study, i.e., RT-PCR results, antigen results, and virus viability, and to observe the development, especially, in people whose AGT returned “false negative” results according to RT-PCR.

Several objections can be raised to the statement above. The first objection that comes into mind is the generally accepted premise that antigen testing is just not sensitive enough for reliable detection in the early stages of the disease. However, it could be the other way around—RT-PCR being “too sensitive for our own good.” It is well-known that dead viral particles that can be detected by RT-PCR are present on the mucosa long after the cessation of infectiousness [9,13]. It is also possible that RT-PCR can also detect dead viral particles inactivated by the mucosal immunity of healthy patients (which is a likely scenario in this case)—in other words, PCR can detect also patients who have been exposed to the virus but have not been infected. In the current situation when lockdown is imposed in many countries with all the societal and economic implications, preventing such people from being needlessly quarantined would be highly beneficial for the society. It is possible that in this respect, using a good and validated AGT could provide more valuable results for the purposes of disease control than PCR testing and could perhaps represent a way of limiting the number of people put into isolation and/or reduce the isolation period. The concept of isolating patients rather on the basis of infectiousness than PCR positivity was previously proposed by Corman et al. [2]. Still, however, this needs to be confirmed by further studies.

Another possible objection is that simply lowering the RT-PCR cycle threshold (or, in the words of a WHO recommendation from January 2021, “careful interpretation of weakly positive results”; [14]) would be a sufficient replacement of the above-proposed approach. This is, of course, a possibility, which has been previously suggested by many, the strongest evidence being brought by Bullard et al. [9]. The analysis of the weakly positive discrepant samples in our study revealed that only a small fraction of samples tested for viability with weakly positive RT-PCR results contained viable virus. However, unlike Bullard who detected no viable virus in samples with Ct > 25 [9], we detected viable virus in several samples with Ct > 30. We must also take into consideration that we only performed viability testing in discrepant samples and that it is likely that among samples that were detected by the AGT as well, the proportion of samples containing viable virus would be much higher. LaScola et al. [15] also detected viable virus in patients with Ct over 30 (as much as 50% in patients with Ct 32), although no viable virus was isolated for samples with Ct > 33. For this reason, we hypothesize that a well-performing AGT would yield better results than a simple reduction of the cycle threshold. In addition, we also propose that virus viability testing should be an inherent part of AGT validation rather than choosing low Ct cut-off values as can often be seen in the validation studies [5].

Lastly, and most importantly, we cannot be 100% sure that patients without a viable virus in the nasopharynx do not shed viable virus from the throat or other parts of the upper respiratory tract (please note that we are still discussing the screening of asymptomatic or mildly symptomatic patients only, i.e., patients without the serious involvement of the lower respiratory tract). Nevertheless, Wölfel et al. [13] found no difference in the PCR loads between the swabs from the throat and nasopharynx in the early stages of the disease, which implies that it is unlikely that significant difference would be found in the virus viability in both regions. Therefore, it is possible, although needs further verification, that such patients do not spread the viable virus during normal activities, sneezing, or a mild cough originating in the upper respiratory tract.

Well-performing AGTs could also possibly serve for termination of isolation in patients with SARS-CoV-2. It is well-known that a week to 8 days after the onset of symptoms, no virus isolation was successful in patients with mild course of the disease [9,13,16], although they still remained RT-PCR positive. Antigen testing could represent a suitable alternative for determining whether or not such a patient needs to remain in quarantine, rather than RT-PCR.

Limitations of this study include the uneven number of patients tested by individual AGTs and, in particular, the low number of samples tested using the Standard Q test. This was, however, given by the numbers of tests provided to us by the Ministry of Health. Another limitation was that only nasopharyngeal swabs were performed. However, we opted for using the same technique that was used for the collection of samples for RT-PCR to be able to directly compare these two methods. Lastly, we did not perform viability testing in all samples but only in the discrepant ones. We concede that knowing the virus viability in all samples would be a great help for understanding the performance of AGT and RT-PCR; however, performing viability testing in more than 1100 samples was just not practically feasible. Still, we believe that such a study (or at least a study measuring viability on a higher number of high Ct samples, not just discrepant ones) should be performed and is already long overdue.

## 5. Conclusions

Our results show that, when using suitable antigen tests, viable virus was present only in approx. 12% of the antigen test “false negatives” (when compared to RT-PCR only). Correcting on that, the best of the evaluated tests yielded 96.4% sensitivity, 97% specificity, and perhaps most importantly, 98.7% negative predictive value. This leads us to propose that (a) virus viability testing, at least of the discrepant samples, should form a part of AGT evaluation and validation process; (b) we propose that antigen testing using a well-performing AGT could represent a valid tool for infection control in high prevalence settings, and (c) that more detailed research on the patients who are “missed” by (well-performing) AGT is needed with respect to their infectiousness to determine whether or not they pose a threat from the perspective of disease spreading.

## Figures and Tables

**Table 1:** Basic description of the patient group.

	Antigen Test				
	Ecotest	JoysBio	ND COVID	Standard Q	VIVADiag
N	318	225	191	139	268
Of which RT-PCR positive	107	90	77	42	91
Women/Men (%)	38.4/61.6	55.1/44.9	53.4/46.6	51.8/48.2	53.4/46.6
Mean age (years) ± SD	45 ± 14	43 ± 15	42 ± 15	42 ± 17	43 ± 15
Proportion (%) * of symptomatic patients	42.8	60.3	52.1	47.8	56.9

* In 14 patients (1.2%), this information was not recorded.

**Table 2:** Agreement between the antigen tests (AGT) and RT-PCR, false negative and false positive samples out of the total number of positive/negative samples according to the particular reference method (RT-PCR or RT-PCR + virus viability).

		Ecotest	JoysBio	ND COVID	Standard Q	VivaDiag
AGT and RT-PCR Agreement/Total		285/318 (90%)	185/225 (82%)	118/191 (61%)	122/139 (88%)	208/268 (78%)
AGT False Negatives	vs. RT-PCR	26/107 (24%)	38/90 (42%)	23/77 (30%)	16/42 (38%)	53/91 (58%)
	vs. RT-PCR +viability	3/84 (4%)	4/56 (7%)	8/62 (13%)	4/30 (13%)	9/47 (19%)
	*Viable virus presence* *	*3/26 (12%)*	*4/38 (11%)*	*8/23 (35%)*	*4/16 (25%)*	*9/53 (17%)*
AGT False Positives	vs. RT-PCR	7/211 (3%)	2/135 (1%)	50/114 (44%)	1/97 (1%)	7/177 (4%)
	vs. RT-PCR + viability	7/234 (3%)	2/169 (1%)	50/129 (39%)	1/109 (1%)	7/221 (3%)

* Samples containing viable virus out of false-negative AGT results when compared to RT-PCR only.

**Table 3:** Screening test performance metrics for individual tests when comparing antigen testing (AGT) results to RT-PCR and when comparing AGT results to RT-PCR corrected on the results of virus viability testing.

		Ecotest	JoysBio	ND COVID	Standard Q	VivaDiag
	Manufacturer- Declared Sensitivity/Specificity	97.7/99.1	98.1/99.2	>95/>95	87.8–91.9/97.6–99.7	90.9/99.1
AGT vs. RT-PCR Only	*Prevalence (PCR)*	*33.6 (28.5–39.1)*	*40.0 (33.5–46.7)*	*40.3 (33.3–47.6)*	*30.2 (22.7–38.6)*	*34.0 (28.3–40)*
	**Sensitivity**	**75.7 (66.5–83.5)**	**57.8 (46.9–68.1)**	**70.1 (58.6–80)**	**61.9 (45.6–76.4)**	**41.8 (31.5–52.6)**
	**Specificity**	**96.7 (93.3–98.7)**	**98.5 (94.8–99.8)**	**56.1 (46.5–65.4)**	**99.0 (94.4–100)**	**96.0 (92.0–98.4)**
	PPV	92.0 (84.3–96.7)	96.3 (87.3–99.5)	51.9 (41.9–61.8)	96.3 (81–99.9)	84.4 (70.5–93.5)
	NPV	88.7 (83.9–92.5)	77.8 (70.8–83.8)	73.6 (63–82.4)	85.7 (77.8–91.6)	76.2 (70.1–81.7)
	ACC	89.6 (85.7–92.7)	82.2 (76.6–87.0)	61.8 (54.5–68.7)	87.8 (81.1–92.7)	77.6 (72.1–82.5)
AGT vs. RT-PCR + viAbility	*Prevalence* *(PCR + Viability)*	*26.4 (21.7–31.6)*	*24.9 (19.4–31.1)*	*32.5 (25.9–39.6)*	*21.6 (15.1–29.4)*	*17.5 (13.2–22.6)*
	**Sensitivity**	**96.4 (89.9–99.3)**	**92.9 (82.7–98)**	**87.1 (76.1–94.3)**	**86.7 (69.3–96.2)**	**80.9 (66.7–90.9)**
	**Specificity**	**97.0 (93.9–98.8)**	**98.8 (95.8–99.9)**	**61.2 (52.3–69.7)**	**99.1 (95–100)**	**96.8 (93.6–98.7)**
	PPV	92.0 (84.3–96.7)	96.3 (87.3–99.5)	51.9 (41.9–61.8)	96.3 (81–99.9)	84.4 (70.5–93.5)
	NPV	98.7 (96.2–99.7)	97.7 (94.1–99.4)	90.8 (82.7–95.9)	96.4 (91.1–99)	96.0 (92.5–98.1)
	ACC	96.9 (94.3–98.5)	97.3 (94.3–99.0)	69.6 (62.6–76.1)	96.4 (91.8–98.8)	94.0 (90.5–96.5)

PPV: positive predictive value; NPV: negative predictive value; ACC: accuracy; numbers in brackets denote 95% confidence intervals.

**Table 4:** Test parameters for individual ATGs for a subgroup of symptomatic patients (estimates and 95% confidence intervals).

		Ecotest	JoysBio	ND COVID	Standard Q	VIVADiag
N/POS_PCR_/POS_PCR+V_		134/86/73	132/76/50	99/58/50	66/33/24	152/73/40
**PCR**	*Prevalence*	*64.2 (55.4–72.3)*	*57.6 (48.7–66.1)*	*58.6 (48.2–68.4)*	*50.0 (37.4–62.6)*	*48.0 (39.9–56.3)*
	Sensitivity	83.7 (74.2–90.8)	60.5 (48.6–71.6)	77.6 (64.7–87.5)	63.6 (45.1–79.6)	46.6 (34.8–58.6)
	Specificity	95.8 (85.7–99.5)	96.4 (87.7–99.6)	56.1 (39.7–71.5)	97 (84.2–99.9)	97.5 (91.2–99.7)
	PPV	97.3 (90.6–99.7)	95.8 (85.7–99.5)	71.4 (58.7–82.1)	95.5 (77.2–99.9)	94.4 (81.3–99.3)
	NPV	76.7 (64.0–86.6)	64.3 (53.1–74.4)	63.9 (46.2–79.2)	72.7 (57.2–85)	66.4 (57–74.9)
**PCR + viability**	*Prevalence*	*54.5 (45.7–63.1)*	*37.9 (29.6–46.7)*	*50.5 (40.3–60.7)*	*36.4 (24.9–49.1)*	*26.3 (19.5–34.1)*
	Sensitivity	98.6 (92.6–100.0)	92 (80.8–97.8)	90 (78.2–96.7)	87.5 (67.6–97.3)	85.0 (70.2–94.3)
	Specificity	96.7 (88.7–99.6)	97.6 (91.5–99.7)	63.3 (48.3–76.6)	97.6 (87.4–99.9)	98.2 (93.7–99.8)
	PPV	97.3 (90.6–99.7)	95.8 (85.7–99.5)	71.4 (58.7–82.1)	95.5 (77.2–99.9)	94.4 (81.3–99.3)
	NPV	98.3 (91.1–100.0)	95.2 (88.3–98.7)	86.1 (70.5–95.3)	93.2 (81.3–98.6)	94.8 (89.1–98.1)

N—number of patients in the group; POS_PCR_—number of positive patients acc. to RT-PCR; POS_PCR+V_—number of positive patients according to the combined RT-PCR+viability endpoint; PPV—positive predictive value; NPV—negative predictive value.

**Table 5:** Test parameters for individual ATGs for a subgroup of asymptomatic patients (estimates and 95% confidence intervals).

		Ecotest	JoysBio	ND COVID	Standard Q	VIVADiag
N/POS_PCR_/POS_PCR+V_		179/20/10	87/13/6	91/19/12	72/8/5	115/17/7
PCR	*Prevalence*	*11.2 (7.0–16.7)*	*14.9 (8.2–24.2)*	*20.9 (13.1–30.7)*	*11.1 (4.9–20.7)*	*14.8 (8.9–22.6)*
	Sensitivity	40 (19.1–63.9)	46.2 (19.2–74.9)	47.4 (24.4–71.1)	50.0 (15.7–84.3)	23.5 (6.8–49.9)
	Specificity	96.9 (92.8–99)	100 (95.1–100)	56.9 (44.7–68.6)	100 (94.4–100)	94.9 (88.5–98.3)
	PPV	61.5 (31.6–86.1)	100 (54.1–100)	22.5 (10.8–38.5)	100 (39.8–100)	44.4 (13.7–78.8)
	NPV	92.8 (87.7–96.2)	91.4 (83–96.5)	80.4 (66.9–90.2)	94.1 (85.6–98.4)	87.7 (79.9–93.3)
PCR + viability	*Prevalence*	*5.6 (2.7–10.0)*	*6.9 (2.6–14.4)*	*13.2 (7.0–21.9)*	*6.9 (2.3–15.5)*	*6.1 (2.5–12.1)*
	Sensitivity	80.0 (44.4–97.5)	100 (54.1–100)	75.0 (42.8–94.5)	80.0 (28.4–99.5)	57.1 (18.4–90.1)
	Specificity	97 (93.2–99.0)	100 (95.5–100)	60.8 (49.1–71.6)	100 (94.6–100)	95.4 (89.5–98.5)
	PPV	61.5 (31.6–86.1)	100 (54.1–100)	22.5 (10.8–38.5)	100 (39.8–100)	44.4 (13.7–78.8)
	NPV	98.8 (95.7–99.9)	100 (95.5–100)	94.1 (83.8–98.8)	98.5 (92.1–100)	97.2 (92–99.4)

N—number of patients in the group; POS_PCR_—number of positive patients acc. to RT-PCR; POS_PCR+V_—number of positive patients according to the combined RT-PCR+viability endpoint; PPV—positive predictive value; NPV—negative predictive value.

## Data Availability

The dataset is available from Figshare at https://doi.org/10.6084/m9.figshare.14096648.v1.

## Appendix A

**Table A1:** Detailed comparison of RT-PCR and antigen test (AGT) for individual tests stratified by cycle threshold (Ct): Strongly positive (Ct ≤ 20), Positive (20 < Ct ≤ 30), Weakly positive (30 < Ct ≤ 40), and Negative (Ct > 40). For better orientation, true positive and true negative values for individual tests are highlighted in grey.

	PCR Result	Patients (N)	ATG Result		Discrepancies N (%)	
			ATG Positive	ATG Negative		of Which Viable Virus ^3^
			N (%) ^1^	N (%) ^1^	N (%) ^2^	
**Ecotest**	**PCR-positive**	**107**	**81 (76%)**	**26 (24%)**	**26 (79%)**	**3 (12%)**
	Strongly positive	5	5 (100%)	0 (NA)	0 (0%)	0 (N/A)
	Positive	56	54 (96%)	2 (4%)	2 (6%)	2 (100%)
	Weakly positive	46	22 (48%)	24 (52%)	24 (73%)	1 (4%)
	**PCR-negative**	**211**	**7 (3%)**	**204 (97%)**	**7 (21%)**	**0 (0%)**
	***Total***	***318***	***88 (28%)***	***230 (72%)***	***33***	
**JoysBio**	**PCR-positive**	**90**	**52 (58%)**	**38 (42%)**	**38 (95%)**	**4 (11%)**
	Strongly positive	2	2 (100%)	0 (0%)	0 (0%)	0 (N/A)
	Positive	43	38 (88%)	5 (12%)	5 (13%)	3 (60%)
	Weakly positive	45	12 (27%)	33 (73%)	33 (82%)	1 (3%)
	**PCR-negative**	**135**	**2 (1%)**	**133 (99%)**	**2 (5%)**	**0 (0%)**
	***Total***	***225***	***54 (24%)***	***171 (76%)***	***40***	
**ND COVID**	**PCR-positive**	**77**	**54 (70%)**	**23 (30%)**	**23 (37%)**	**8 (35%)**
	Strongly positive	0	0 (NA)	0 (NA)	0 (0%)	0 (N/A)
	Positive	36	30 (83%)	6 (17%)	6 (8%)	6 (100%)
	Weakly positive	41	24 (59%)	17 (41%)	17 (23%)	2 (12%)
	**PCR-negative**	**114**	**50 (44%)**	**64 (56%)**	**50 (68%)**	**0 (0%)**
	***Total***	***191***	***104 (54%)***	***87 (46%)***	***73***	
**Standard Q**	**PCR-positive**	**42**	**26 (62%)**	**16 (38%)**	**16 (94%)**	**4 (25%)**
	Strongly positive	1	1 (100%)	0 (NA)	0 (0%)	0 (N/A)
	Positive	22	18 (82%)	4 (18%)	4 (24%)	3 (75%)
	Weakly positive	19	7 (37%)	12 (63%)	12 (71%)	1 (8%)
	**PCR-negative**	**97**	**1 (1%)**	**96 (99%)**	**1 (6%)**	**0 (0%)**
	***Total***	***139***	***27 (19%)***	***112 (81%)***	***17***	
**VIVA diag**	**PCR-positive**	**91**	**38 (42%)**	**53 (58%)**	**53 (88%)**	**9 (17%)**
	Strongly positive	0	0 (0%)	0 (NA)	0 (0%)	0 (N/A)
	Positive	39	27 (69%)	12 (31%)	12 (20%)	7 (58%)
	Weakly positive	52	11 (21%)	41 (79%)	41 (68%)	2 (5%)
	**PCR-negative**	**177**	**7 (4%)**	**170 (96%)**	**7 (12%)**	**0 (0%)**
	***Total***	***268***	***45 (17%)***	***223 (83%)***	***60***	

^1^ percentage of the reference RT-PCR result; ^2^ percentage of discrepancies between RT-PCR and AGT in the respective group; ^3^ percentage of samples with viable virus out of the discrepant results in the respective group.
